# Inter relationship between some routine semen evaluation parameters in Jersey X local hill cattle crossbred bulls

**Published:** 2012-05-01

**Authors:** M. Sharma, M. Singh, S. Kapoor, S. Jasial

**Affiliations:** 1 *Veterinary officer, Veterinary Hospital Khajjiar, Chamba (H.P.) India*; 2 *Department of Veterinary Gynaecology and Obstetrics, College of Veterinary and Animal Sciences, Himachal Pradesh Agricultural University, Palampur (H.P.) India*; 3 *Veterinary Officer, Sperm Station, Palampur (H.P.) India*

**Keywords:** Acrosome, Crossbred bulls, HOST, Semen

## Abstract

The present study was conducted with an objective of establishing a relationship between various routine semen evaluation parameters. Work was conducted at Sperm Station Palampur, Himachal Pradesh, on the semen from five Jersey X local hill cattle crossbred breeding bulls. A total of 40 ejaculates (8 from each bull), were analysed at five different stages of processing namely post dilution, post equilibration, post thaw and after 1 and 2 hours incubation post thaw at 37°C for progressive motility, live dead count, reaction to hypo-osmotic solution, acrosomal integrity and gross morphological abnormalities. The results of the study revealed a highly significant (P<0.01) correlation between the various semen evaluation parameters except for the gross morphological abnormalities where there was a significant (P<0.05) negative correlation between the acrosomal integrity and gross morphological abnormalities.

## Introduction

Artificial Insemination (AI) is a reproductive technology that has made possible the effective use of best breeding males, thus greatly improving the genetic quality of breeding herds (Januskauskas and Zilinskas, 2002). The success of AI depends upon the ability to screen for semen with high fertilization potential (Holt *et al.*, 2007).

Thus, given the biological and economic importance of knowing with certainty the potential fertility of the semen for AI before insemination, it becomes essential to explore aspects that relate to fertility. Although the most conclusive evidence of fertility from freeze-thawed semen is made on the basis of pregnancy rate in the females served, semen evaluation offers predictive information on expected performance of the male.

It has been generally accepted that there is some relation between the fertility of the semen and its measurable properties (Januskauskas and Zilinskas, 2002).

Screening of the semen at initial stages allows elimination of poor quality semen while the proper assessment of the post-thaw quality of spermatozoa can provide insights into the fertilizing capacity of the cryopreserved spermatozoa (Januskauskas and Zilinskas, 2002).

So, a number of laboratory evaluation tests measuring the physical and functional integrity of spermatozoa *in-vitro* have been devised over the past few decades. Keeping in view the economic and time constraints in field conditions, routine semen evaluation tests like sperm viability, progressive motility, hypo-osmotic swelling tests, acrosomal integrity and morphological abnormalities have been evaluated that are rapid, relatively inexpensive and easily executable.

In terms of prediction, if two variables are correlated perfectly, then knowing the value of one score permits a perfect prediction of the score on the second variable. Generally, whenever two variables are significantly correlated, the researcher may use the score on one variable to predict the score on the second (Ho, 2006).

The present study was conducted with an objective of establishing a relationship between the various routine semen evaluation parameters and drawing equations for the estimation of various seminal attributes on the basis of one parameter.

The aim was to simplify the evaluation process by using the correlation equations to work out the related routine assays instead of evaluating all of them.

## Materials and Methods

Work was conducted at Sperm Station Palampur, India (32.6°N, 76.3°E, altitude 1290.8 m) on the semen from five Jersey X local hill cattle crossbred breeding bulls.

The bulls chosen for the study were reared under identical conditions and were known to donate consistently good quality semen.

Semen was collected twice a week by artificial vagina method. The collected semen was subjected to initial examination of volume, colour, concentration and initial motility qualifying after which they were further processed as per standard laboratory procedures. The minimum initial standards were a volume of 2 ml, colour ranging from milky white to creamy, minimum concentration of 500 million/ml and an initial motility of above 70 percent.

The selected ejaculate was diluted with tris egg yolk extender to attain a final concentration of 80 million/ml after which they were filled into French mini straws, sealed and labelled at 34°C. The semen in the straws were allowed to equilibrate at 4°C for about 4 hours and then shifted to programmable biofreezers where the temperature was further brought down to -140°C using liquid nitrogen vapours. These straws were finally shifted to liquid nitrogen containers and stored till use.

Eight ejaculates from each bull (total 40 ejaculates) were analysed for percent viability (Buttle *et al.*, 1965), progressive motility, reaction to 150 mOsmol hypo-osmotic solution (Pant *et al.*, 2002), acrosomal integrity (Watson, 1975) and morphological abnormalities (Blom, 1973) at five stages of processing namely post-dilution (34°C), post-equilibration (4°C), post-thaw (37°C) and 1 hr and 2 hrs incubation post-thaw at 37°C.

Thawing of frozen semen straws for post thaw evaluation was done in water bath at 37°C for 30 seconds.

### Viability

To ascertain the percentage of live spermatozoa, a smear semen mixed (1 drop) with eosine-nigrosine (5 drops) (Eosin 1.67gm, Nigrosin 10 gm, Distilled water 100 ml) was examined under high power (40X) objective.

All stained and partially stained spermatozoa were considered dead. The percentage of live spermatozoa was determined by counting at least 200 spermatozoa.

### Progressive motility

Progressive motility was analysed by examining three random fields under the high power (40X) objective of a warm stage (37°C) phase contrast microscope (Nikon Eclipse E 400, Japan) for the sperms that were not showing progressive motility (i.e. sperms with circular, backward and oscillating movements).

This was followed by heat killing the sperm and counting the total number of sperm in three random fields. The percentage of progressively motile sperm was calculated as:

([Total sperm - sperm counted before passing over flame] / Total sperm) X 100.

### Hypo-osmotic swelling test (HOST)

One ml of pre incubated hypo-osmotic solution (100 mOsm/L) was mixed with 0.1 ml of extended semen in a small test tube. A control was set by mixing 1 ml of control solution (300 mOsm/L) with 0.1 ml of same semen in another test tube. Both test tubes were then incubated in water bath at 37°C for 30 minutes. A drop from each solution of incubated semen was examined under phase contrast microscope at 400x magnification for swelling (ballooning or curling) of sperm tails. A minimum of 100 spermatozoa were counted.

The proportion of swollen spermatozoa in the control sample was subtracted from the proportion of swollen spermatozoa in hypo-osmotic solution. The resultant figure was considered as percentage of HOST reactive spermatozoa.

### Acrosomal integrity

For evaluation of acrosomal integrity dried smears fixed in formalin were stained with giemsa overnight and evaluated under oil immersion to evaluate the acrosomal defects by counting at least 200 sperm. Acrosomal defects were further classified as swollen, ruffled, abnormal contour and detached acrosomes.

### Morphological abnormalities

The semen smears were stained with Rose Bengal (Rose Bengal 3g, Commercial formalin 1 ml, Distilled water 100ml) and the percentage of abnormal sperm was determined by examining 200 sperm under oil immersion objective. Head, neck, mid-piece and tail abnormalities were classified according to Blom (1973).

### Statistical analysis

The data obtained was analysed using SAS statistical package version 9.2.

Generalized Linear Model of one way ANOVA based on Fisher’s Least Significant Difference method was used to determine the individual bull variation with regard to the various seminal attributes at different stages of processing. Multivariate analysis was used to determine correlations and to frame regression equations and their significance was tested again by using ANOVA.

## Results

The semen was evaluated at five different stages to analyse the variation in the parameters at various stages of processing.

The parameters were statistically analysed separately at all the five stages of processing to determine the correlation between them.

All the parameters were found to have significant correlations (positive/negative) except for the gross morphological abnormalities that were not correlated to any of the parameters. Since, the various parameters were found to have similar correlations at different stages of semen processing, data of all the stages for a particular parameter were pooled to obtain a larger sample size (n=200) and were then subjected to statistical analysis.

**Fig. 1:**
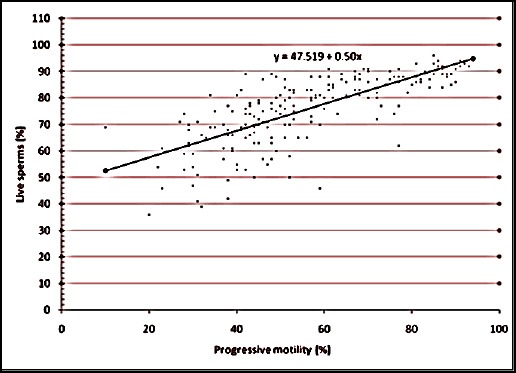
Relationship between live sperms and progressive motility of spermatozoa of Jersey X local hill cattle crossbred bulls.

**Fig. 2:**
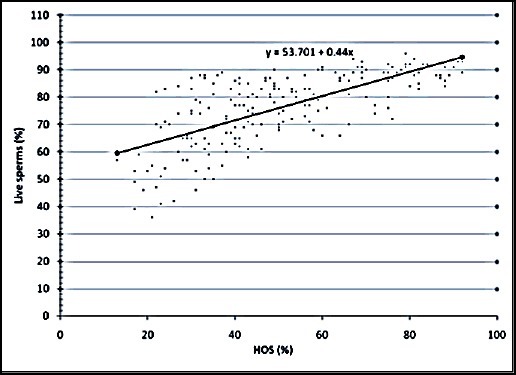
Relationship between live sperms and HOS activity of spermatozoa of Jersey X local hill cattle crossbred bulls.

**Fig. 3:**
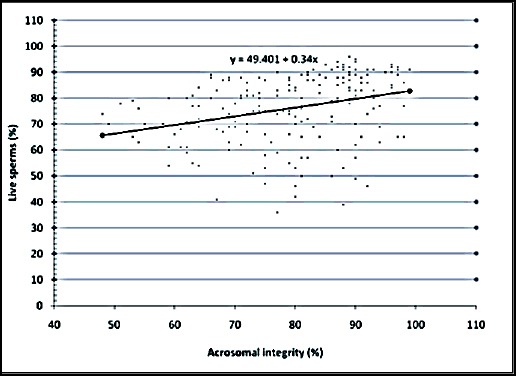
Relationship between live sperms and acrosomal integrity of spermatozoa of Jersey X local hill cattle crossbred bulls.

**Fig. 4:**
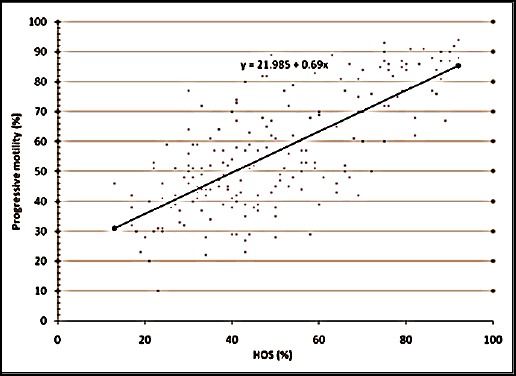
Relationship between progressive motility and HOS activity of spermatozoa of Jersey X local hill cattle crossbred bulls.

**Fig. 5:**
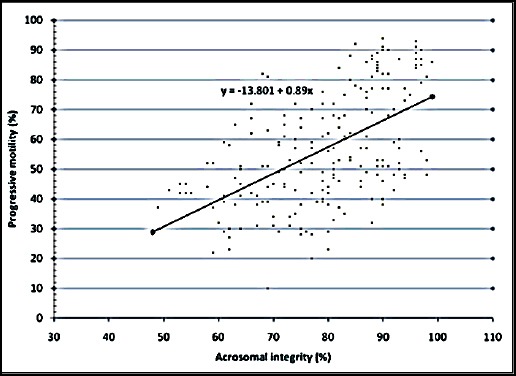
Relationship between progressive motility and acrosomal integrity of spermatozoa of Jersey X local hill cattle crossbred bulls.

**Fig. 6:**
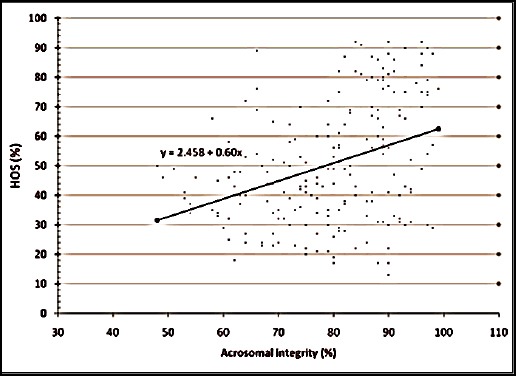
Relationship between HOS activity and acrosomal integrity of spermatozoa of Jersey X local hill cattle crossbred bulls.

The relationship between the various semen evaluation parameters along with their regression equations are shown in Figures 1 to 6 and Table 1.

**Table 1:** Inter-relationship between semen evaluation parameters in Jersey X local hill cattle crossbred bulls.

No.	Relationship between parameters	Correlation coefficient	Regression estimate	Regression equation
1	Viability with Progressive motility	0.744**	0.504±0.03	y = 47.519 + 0.50x
2	Viability with HOST	0.695**	0.444±0.03	y = 53.701 + 0.44x
3	Viability with acrosomal integrity	0.311**	0.337±0.07	y = 49.401 + 0.34x
4	Viability with morphological abnormalities	0.068 NS	0.198±0.21	y = 74.744 + 0.20x
5	Progressive motility with HOST	0.730**	0.689±0.04	y = 21.985 + 0.69x
6	Progressive motility with acrosomal integrity	0.556**	0.891±0.09	y = -13.801 + 0.89x
7	Progressive motility with morphological abnormalities	0.020 NS	0.087±0.31	y = 56.163 + 0.09x
8	HOST with acrosomal integrity	0.357**	0.606±0.11	y = 2.458 + 0.60x
9	HOST with morphological abnormalities	0.123 NS	1.023±0.32	y = 43.342 + 1.02x
10	Acrosomal integrity with morphological abnormalities	-0.178*	-0.483±0.19	y = 82.582 - 0.48x

* (P<0.05),

** (P<0.01), NS (not significant).

## Discussion

The quality of semen has been assessed by various conventional evaluation parameters. The relationship between different semen evaluation parameters is important, since on the basis of evaluation of one parameter, a fair idea of other parameters can be formulated. This relationship is expected as the sperm plasma membrane is a continuous structure covering the head, mid-piece and the tail (Karp, 2009).

Almost all the parameters evaluated in the study are related to plasma membrane integrity at one point or the other. Membrane integrity and the stability of its semi-permeable features are prerequisites for the viability of spermatozoon. Furthermore, if the plasmalemma is intact but functionally unstable, the spermatozoon is not capable of interacting with its environment and thus, is unable to fertilize (Rodriguez-Martinez, 2007).

The results of the present study were in agreement with some previous work on cattle (Correa and Zavos, 1994; Kumar, 2004; Lodhi *et al.*, 2008), human (Jeyendran *et al.*, 1984), equine (Mantovani *et al.*, 2002), ram (Bohlooli *et al.*, 2012) and fresh goat spermatozoa (Fonseca *et al.*, 2005; Nur *et al.*, 2005). The correlation that was recorded between motility, viability, acrosomal integrity and HOST was expected since they are all related to plasma membrane integrity (Brito *et al.*, 2003).

Furthermore, the correlation between the membrane integrity and motility may be attributed to the fact that motility is a function of intracellular adenosine triphosphate (ATP) content (Januskauskas and Rodriguez-Martinez, 1995). Therefore, rapid leakage of intracellular ATP through the damaged sperm plasma membrane due to death or anisosmotic condition is certain to affect sperm motility (Bohlooli *et al.*, 2012).

Higher correlation between motility and HOST was probably due to the fact that both analysed the integrity of the tail membrane. Further, this correlation was even stronger between viability and HOST due to increased probability of errors associated with measurement of progressive motility (Saacke *et al.*, 1991; Stalhammar *et al.*, 1994; Garner, 1997; Christensen *et al.*, 1999).

Similar findings have been reported earlier for sperm viability and progressive motility (Singh, 1986; Vyas *et al.*, 1992; Singh and Pant, 1999; Januskauskas *et al.*, 1999) and also between HOST and viability (Correa and Zavos, 1994).

Similarly, in some earlier studies, correlation has been established between sperm motility, live sperm percentage and HOST reactive spermatozoa (Kumar, 2004; Lodhi *et al.*, 2008) and also between percentages of live sperms and acrosome intact sperms with percentage of motile sperms (Kirk *et al.*, 2005). Contrary to our findings, low, moderate or non-significant correlation was previously reported between HOST responsive spermatozoa and plasmalemma intact sperms identified by vital stain (Esteves *et al.*, 1996; Neild *et al.*, 1999; Brito *et al.*, 2003). However, in this study, the proportion of viable sperms identified by HOST was consistently lower than the proportion of viable spermatozoa identified by vital stains. This result is consistent with the concept that vital stains are used to evaluate physical plasmalemma damage, while HOST evaluates biochemical activity of plasmalemma; that plasmalemma is intact does not ensure that it is functional (Brito *et al.*, 2003).

Negative correlations of these parameters with morphological abnormalities at various stages of processing have also been reported (Vyas *et al.*, 1992) but no significant relationship of the gross morphological abnormalities could be established with any other evaluation parameters except for acrosomal integrity in this study. This can be attributed to the system of classification of the abnormalities. The relationship with acrosomal integrity may be ascribed to the association of acrosomal abnormalities to head abnormalities.
